# Overcome Cancer Cell Drug Resistance Using Natural Products

**DOI:** 10.1155/2015/767136

**Published:** 2015-09-01

**Authors:** Pu Wang, Hua Li Yang, Ying Juan Yang, Lan Wang, Shao Chin Lee

**Affiliations:** School of Life Sciences, Shanxi University, Taiyuan, Shanxi 030006, China

## Abstract

Chemotherapy is one of the major treatment methods for cancer. However, failure in chemotherapy is not uncommon, mainly due to dose-limiting toxicity associated with drug resistance. Management of drug resistance is important towards successful chemotherapy. There are many reports in the Chinese literature that natural products can overcome cancer cell drug resistance, which deserve sharing with scientific and industrial communities. We summarized the reports into four categories: (1) *in vitro* studies using cell line models; (2) serum pharmacology; (3) *in vivo* studies using animal models; and (4) clinical studies. Fourteen single compounds were reported to have antidrug resistance activity for the first time. *In vitro*, compounds were able to overcome drug resistance at nontoxic or subtoxic concentrations, in a dose-dependent manner, by inhibiting drug transporters, cell detoxification capacity, or cell apoptosis sensitivity. Studies *in vivo* showed that single compounds, herbal extract, and formulas had potent antidrug resistance activities. Importantly, many single compounds, herbal extracts, and formulas have been used clinically to treat various diseases including cancer. The review provides comprehensive data on use of natural compounds to overcome cancer cell drug resistance in China, which may facilitate the therapeutic development of natural products for clinical management of cancer drug resistance.

## 1. Introduction

Cancer is one of the most common noncommunicable diseases and one of the leading causes of death worldwide, with approximately 14 million new cases and 8.2 million cancer-related deaths in 2012, which are expected to rise in the future [1]. In China, according to the Annual Cancer Registry in 2013, more than 3 million new cases were diagnosed with cancer, which is equivalent to 6 patients diagnosed per minute, and the prevalence has been increasing. This casts great socioeconomic burdens. Despite the fact that chemotherapy is central to clinical management of cancer, failure in chemotherapy is not uncommon, mainly due to the dose-limiting toxicities, which is associated with the occurrence of drug resistance. Cancer cell drug resistance occurs in inherited or acquired manners. Inherited drug resistance is the initial unresponsiveness of cancer to a specific drug, while acquired resistance refers to emerged drug resistance after initial successful drug treatment. No matter whether it occurs before or after chemotherapy, how to overcome drug resistance is a key issue for cancer management, which has been under investigation. Taking nanomedicine, for example, scientists have been trying to deliver drugs to cancer cells by nanoparticles, which may provide an approach to overcome drug resistance [2], as nanoparticles are hoped to enhance drug permeability, retention, and intracellular accumulation as well as increase drug circulating time. Amongst various nanoparticles, ThermoDox is currently under phase III clinical trial, which is a type of lipid-based nanoparticle that uses polyethylene glycol to increase circulation time. There are also clinical trials that use dendrimers to deliver chemodrug to treat different cancers such as breast cancer, non-small cell lung cancer, and advanced pancreatic cancer. Apart from the modern technologies, use of chemical compounds to counteract drug resistance is another strategy, for which natural products may be useful. Indeed, natural products provide a useful resource for drug development. About 36% of the small molecule compounds approved by US Food and Drug Administration between 1999 and 2008 are natural products or their derivatives [3]. In the past years, there is an emerging view that targeted therapies are superior to traditional chemotherapy, which seems to promote the development of targeted therapy, leaving the therapeutic development of natural compounds more or less being ignored. Unfortunately, the drawbacks of targeted therapy start to appear after years in practice [4]. Majority of cancers are not targetable, as they show disturbances in multiple oncogenic pathways and adaptive mechanisms that limit the efficacy of targeted therapy, and the treatment benefit using targeted therapy is often less than 6 months. Combination treatment using multiple targeted therapeutic agents may not be better; it can have greater unacceptable toxicity than clinical benefits. Thus, the natural products have come back to the spot light in the area of cancer management; 12 natural products and derivatives have been approved for cancer treatment since 2007. Some natural compounds such as quercetin and curcumin are under clinical trials to overcome cancer drug resistance [5]. In China, in particular, possibly due to the long history of using natural compounds in clinical management of disease including cancer, as is practiced in Traditional Chinese Medicine, there are many reports on overcoming cancer drug resistance using natural compounds. These investigations have produced abundant informative data, which are unlikely to be commonly known to other scientific and industrial communities. Sharing the information is likely to be appreciated.

This review summarizes and discusses the studies on the use of natural products to overcome cancer drug resistance, which were published in the Chinese literature. These studies are organized into four categories: (1)* in vitro* studies using various cell line models; (2) serum pharmacology studies that test the antidrug resistance effects of serum samples from animals fed with natural products; (3)* in vivo* studies that use animal models bearing tumor xenografts or transplantable tumor cells; and (4) clinical studies that give natural products to patients in addition to conventional chemotherapy, in comparison with patients who receive conventional chemotherapy alone.

We are aware that there are many excellent works in this area published in English. They have been reviewed, including a review by Cort and Ozben this year [6]. There are also Chinese authors who publish reports in English rather than Chinese journals. For example, Zhang and coworker show that a synthetic derivative of 23-hydroxybetulinic acid can reverse cancer cell multidrug resistance [7]. Guo et al. note that *β*-Elemene can reverse multidrug resistance mediated by the ABCB1 transporter [8]. Moreover, marine drugs can provide promising candidates for reversal of cancer cell drug resistance, such as lamellarin O [9] and sipholenol A [10]. These informative data are not included in this review.

## 2. Mechanisms of Drug Resistance

**Figure 1:**
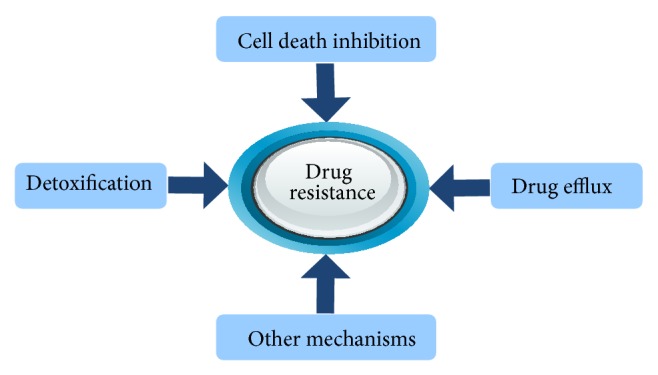
Mechanisms underlying the development of cancer cell drug resistance.

Cancer chemoresistance occurs through various biological mechanisms that include drug efflux, inhibition of cell death, detoxification, DNA damage repair, alternation of drug targets, stem cells, and epithelial to mesenchymal transition [11, 12]. Amongst these mechanisms, drug efflux, cell death inhibition, and detoxification are commonly studied, which are relevant to this review (Figure 1).

### 2.1. Drug Efflux

A family of proteins can expel drugs out of cancer cells and decrease the intracellular drug concentration, thereby preventing the drug toxicity. The human genome encodes 48 members of such proteins called ATP-binding cassettes (ABC), which fall into ABCA to ABCG 7 subgroups, with 15 of them being associated with drug resistance. These proteins are believed to be the major players in the development of cancer cell chemoresistance [13]. The best characterized ABC proteins are MDR1 (p-glycoprotein), multidrug resistance-associated proteins (MRPs), and breast cancer resistant protein (BCRP). MDR1 is the first ABC member discovered with a role in drug resistance. It is expressed in nearly all tissues and has been found to mediate drug resistance in many cancers including breast, colon, gastric, kidney, leukemic, liver, and pancreatic cancer [14]. MRPs represent the largest branch of ABC transporters. This subgroup of proteins includes nine efflux transporters (MRPs1–9), one gated chloride channel, and two potassium channel regulators [8], with MRP1 as the founding member. MRP1 is ubiquitously found in a number of human cells, the elevation of which is associated with drug resistance in many types of cancer. BCRP was first cloned from drug resistant human breast cancer cells, whereby it gets the name [15], which is expressed in many types of cells [16].

### 2.2. Inhibition of Cell Death

Apoptosis is the major type of cell death triggered by chemodrugs. There are two established pathways of apoptosis. The intrinsic pathway centers on mitochondria. Apoptotic signals recruit proapoptotic molecules such as Bax to mitochondria, followed by triggering mitochondria to release cytochrome c that leads to activation of downstream caspases which are apoptosis effectors to cause cell damage. There are also antiapoptotic factors that play an important part in the decision of life or death, which include Bcl-2 and Akt. Bcl-2 is located on mitochondria, which can inhibit cytochrome c release. The extrinsic pathway is activated by binding of ligand to death receptor on cell surface, which activates caspase-8 and then downstream caspases. Bcl-2 and Akt as well as NF-*κ*B are found to be highly expressed in some type of cancers, which are associated with drug resistance. Akt itself is a protein kinase which is activated by PI3 kinase. Once activated, Akt can regulate a variety of apoptosis signal mediators to inhibit apoptosis. It can phosphorylate I*κ*B, which results in NF*κ*B activation that promotes survival and Bad phosphorylation/inactivation to block apoptotic signal. In addition, phosphorylation of caspase-9 by Akt also blocks the induction of apoptosis, and both Akt and NF*κ*B can activate Bcl-2 to inhibit cytochrome c release from mitochondria [17]. Suppression of proapoptotic proteins such as Bax is also associated with the occurrence of drug resistance. In the clinical setting, upregulation of antiapoptotic proteins (i.e., Bcl-2) and downregulation of proapoptotic proteins (i.e., Bax) are found to be associated with poor prognosis of cancer patients [11, 18], which supports a causal role of apoptosis inhibition in the occurrence of cancer cell drug resistance.

### 2.3. Detoxification

GSH-GST system is one of the major detoxification mechanisms, which is in relevance to the development of drug resistance [19]. Levels of GSH and GST activity are in positive correlation with cancer stage [20]. Protein level of GST*π*, in particular, is found to be associated with chemoresistance in patients with different cancers [21–23]. In the clinical setting, GST gene polymorphisms are associated with the development of different cancers [24] and the response of cancer patients to chemotherapy [25]. These observations support a role of GSH-GST system in the regulation of cancer cell response to chemodrugs.

GSH is a small peptide composed of glutamate, cysteine, and glycine. It is an important cellular antioxidant that can suppress oxidative stress to maintain normal cellular redox homeostasis, which relies on its conjugation properties of the sulfhydryl moiety of the cysteine residue. GSH has the ability to directly scavenge cellular reactive oxygen species in a nonenzymatic manner. Alternatively, it serves as a cofactor for GSH peroxidase in the reduction of H_2_O_2_ and other peroxide species. The GSTs are classical phase II metabolic enzymes including GST*π*. They can detoxify xenobiotics via conjugation of reduced GSH with the electrophilic center of a large spectrum of hydrophilic molecules. Although GSH-GST system helps in the maintenance of normal redox homeostasis and the protection against oxidative damage, it has adverse effect in chemotherapy, as it is also intimately involved in the detoxification of numerous xenobiotics. GST family of enzymes utilize GSH as a cofactor in the phase II metabolism of various chemotherapeutic agents, resulting in the formation of GSH-drug conjugates that are more water soluble than their parent compounds and therefore are easier for transporter-mediated efflux. Unfortunately, many chemodrugs are substrates of GSTs. This explains why elevated level/activity of intracellular GSH-GST system is often implicated in the development of cancer cell resistance to various chemodrugs [2].

## 3. Natural Products Overcome Cancer Cell Drug Resistance on Cell Line Models through Various Molecular Mechanisms

Chinese people have been treating diseases using Traditional Chinese Medicine for long, which typically involves the use of natural products in addition to acupuncture, moxibustion, and physiotherapy. Possibly due to this heritage, we are keen to use natural products to overcome cancer cell drug resistance. Wang and coworkers [26] treated bladder cancer T24/ADM cells by doxorubicin with or without osthole. They found that the IC50 of osthole was 76.5 *μ*M and it was not toxic to the cells at the concentrations of 17 *μ*M or below. However, at 17 *μ*M, it was able to lower the IC50 of doxorubicin from 1.0 to 0.4 *μ*M, resulting in an increase of drug sensitivity by 2.5 times. Nong and coworker [27] investigated the antidrug resistance effects of evodiamine using A549/DDP cell line. The compound was not toxic to both resistant and sensitive (A549) cells at the concentrations of 5 *μ*g/mL or below. At 0.25 *μ*g/mL, it lowered the IC50 of cisplatin from 76.7 to 6.7 *μ*g/mL, resulting in an increase in the drug sensitivity of A549/DDP cells by 11.4 times. In mechanistic analysis, the authors found that evodiamine was able to decrease the mRNA levels of MDR1 and Bcl-2 and decrease the level of pIKK*α*, indicating that the compound acts on multiple pathways to overcome drug resistance. Celastrol also has significant antidrug resistance activity. In K562/A02 cells, it can increase the cell sensitivity to chemodrug by 117.9 times, concomitantly with a significant increase in intracellular drug concentration and a decrease in MDR1 protein level [28]. In another study, embelin was found to increase the sensitivity of drug resistant K562 cells to daunorubicin by 7.5 times [29].

There are many more studies using cell line models to test the antidrug resistance activity of natural products. Some studies focus on antidrug resistance effect of compounds without examining the underlying mechanisms (Table 1). Some others investigate the antidrug resistance activity of natural products and the mechanism of drug efflux (Table 2) or inhibition of apoptosis (Table 3). There are natural products that can overcome drug resistance and regulate different pathways, suggesting that they act on multiple molecules to achieve their biological activity (Table 4). Moreover, some compounds are found to target GSH-GST system. These include arsenic trioxide [30], neferine [31], quercetin [32], emodin [33], irisquinone [34], tetramethylpyrazine [35], and ganoderma lucidum polysaccharides [36].

Fourteen single compounds are shown to be able to overcome cancer cell drug resistance for the first time. These include evodiamine, peiminine, isorhynchophylline, berberine, ephedrine, ginsenoside Rb1, oridonin, oxymatrine, methylether-scutellarein, sodium norcantharidate, phenylpropanoid glycoside, retinoic acid, schizandrin A, and baicalin (Tables 1–4), which deserve further investigations. Also for the first time, the studies have revealed the antidrug resistance activities of the various herbal extracts (Tables 1–4).

Some of these compounds (Tables 1–4) are in clinical use for the management of various diseases. For example, oxymatrine is in clinical use to suppress replication of hepatitis B virus [37] and prevent hepatic fibrosis [38]. Berberine is commonly used to treat bacterial and viral infections as well as to increase insulin sensitivity and lower the level of blood sugar [39]. Ephedrine is used to regulate blood pressure [40]. Sodium norcantharidate is used alone or in combination with other established chemotherapeutic drugs to treat cancers [41]. Baicalin is useful in treating psoriasis [42].

Some information has not been included in Tables 1–4, which deserves specification: (1) in the reviewed studies (Tables 1–4), the compounds are commonly used in no-toxic or subtoxic concentrations; (2) the tested compounds can overcome drug resistance in a dose-dependent manner; (3) in some but not all studies, it has been shown that the drug resistance-related genes/proteins are upregulated in drug resistant cells compared to their drug sensitive counterparts, and the tested compounds lower the expression level of the drug resistance-associated genes/proteins in the resistant cells; (4) the tested compounds largely increase drug sensitivity of resistant cells, with no or weak effect on the drug sensitive counterparts, which suggests that the antidrug resistance activity of the compounds has a specificity; (5) some studies have demonstrated the antidrug resistance effects of the natural products on multiple cell line models treated with different chemodrugs; and (6) all of the mentioned herbs and some of the single compounds have been used in clinical settings in China in treating various diseases, which is not further discussed in detail.

## 4. Serum Pharmacology

Compounds are introduced to cell culture in the* in vitro* studies, and their actions may be different when they are applied* in vivo*, since they will be subjected to metabolism in the latter condition. Serum pharmacological study provides a mean to address the issue, at least in part [43]. In this type of assay, animals are fed with compounds or herbal extracts over a short period of time, and serum samples are collected for bioactivity assay* in vitro* on cell line models. The outcomes are more likely to represent the bioactivity of compounds* in vivo*. Usually, compounds are given to animal in an amount equivalent to that used in humans in terms of quantity per unit of body weight. Serum is often applied to cell culture at the concentrations between 5 and 20% in volume. At least 52 serum pharmacology studies are recorded in the Chinese database used for information acquisition, all of which use extract of herbal formulas, except one that uses extract of single herb extract, possibly due to the reason that herbal formulas rather than single herb are commonly used in the practice of Traditional Chinese Medicine. Deng and coworkers [44] investigated the antidrug resistance effect of Changweiqing. They fed rats with Changweiqing extract for 5 days and collected serum samples. In control groups, rats were given saline. The serum samples from the rats in the experimental group were found to be able to increase the sensitivity of drug resistant HCT8/V cells to cisplatin by 13 times* in vitro*. The antidrug resistance effect of Changweiqing serum has also been observed on oral epidermoid carcinoma cell line KB-A-1. It increased the sensitivity of the drug resistant cells to doxorubicin in a dose-dependent manner, which reached 5.2 times at the concentration of 5% in cell culture medium [45]. Wenxia formula is another formula that can overcome drug resistance. Serum from animals fed with this formula extract can increase the sensitivity of A549/DDP cells to cisplatin by 2.5 times [46]. Five more serum pharmacology studies are listed in Table 5. The herbal compositions of these formulas are listed in Table 7. Moreover, these formulas have been used in the clinical setting in China to treat various diseases. Changweiqing, for example, is in clinical use to treat constipation [47], infection of Helicobacter pylori [48], and chronic gastritis [49].

## 5. Overcome Drug Resistance in Animal Models

Since many natural compounds can overcome cancer cell drug resistance* in vitro* (Tables 1–4), it is logical to ask whether the bioactivities can be replicated* in vivo*, that is, in animal models. Indeed, the effects have been shown in a number of studies in animal models. Zhang and coworker [50] built an experimental model of nude mice with xenograft of drug resistant HCT116/L-OHP cells and divided the mice into two groups. One was treated with oxaliplatin, which serves as the control. In the experimental group, mice were treated with oxaliplatin plus Changweiqing extract, 5 times a week for 3 weeks. Compared with the tumor size in the controls, The tumor size in mice treated with Changweiqing was reduced by 3.1 times on average, compared with that in the controls. Feng and coworker [51] treated mice bearing xenograft of drug resistant MCF-7/ADM cells with doxorubicin (control group) or doxorubicin plus extract of Huatan Sanjie formula (experimental group). Herbal extract was applied only twice, at days 8 and 15, respectively. At day 17, mice were sacrificed and tumor tissues were dissected. Compared to the controls, the mice in the experimental group had decreased in tumor tissue weight by 4.8-fold. Interestingly, the tumor tissues from the mice in the experimental group had increased level of doxorubicin and decreased protein level of MDR1. The data, taken together, suggest that the herbal extracts overcome the drug resistance of the tumor xenograft by inhibiting drug pump. In addition to herbal extracts, single compound is found to overcome drug resistance* in vivo*. In the* in vitro* study, epigallocatechin gallate (30 *μ*g/mL) is not toxic to drug resistant KBV200 cells but increases their sensitivity to vincristine by 13 times, increases the intracellular vincristine concentration by 3 times, and reduces the intracellular MDR1 protein concentration. In mice model, the compound significantly increases the antitumor effect of vincristine and decreases the mRNA levels of MRD1 as well as LRP in tumor tissues [52]. Similarly, Puerarin was found to increase the tumor growth inhibition rate from 18.1% (treated with 5-fluorouracil, control group) to 56.7% (treated with 5-fluorouracil plus puerarin, experimental group) and decrease the protein levels of MDR1 and MRP in tumor tissues [53]. In nude mice with tumor xenograft (HCT8/VCR cells), curcumin increases the chemotherapeutic potential of vincristine and decreases the protein levels of MDR1 as well as survivin in the tumor cells [54]. Cepharanthine (4 *μ*M) is not toxic to EAC/ADR cells* in vitro* but can increase the cell sensitivity to doxorubicin by 13 times and inhibit NF-*κ*B activity which is upregulated in the drug resistant cells. In mice model, the compound can increase the life span of mice by 75.4%. Wang [55] developed a mouse liver cancer cell line (Hca cells) model to investigate antidrug resistance effect of cepharanthine hydrochloride. The resistant cells have higher levels of MDR1 and MRP1 compared to their drug sensitive parental cells and are resistant to multiple chemodrugs such as etoposide, daunorubicin, and vincristine. In mice bearing drug resistant Hca cells, cepharanthine hydrochloride can inhibit the expression of the drug transporters and increase the intracellular concentration of chemodrugs as well as life span of animals. In mice bearing drug resistant S180 sarcoma cells, matrine (100 mg/kg a day for 10 days) reduces the protein levels of MDR1, LRP, and Topo II by 78%, 84%, and 65%, respectively [56]. Also in S180-bearing mice, tetrandrine reduces the protein level of MDR1, increases the protein levels of Fas and Trail, and enhances the tumor cell apoptosis [57]. These results indicate that the natural products are potent agents to overcome cancer cell drug resistance* in vivo*, by inhibiting molecules that mediate drug resistance.

## 6. Clinical Studies

The ultimate goal of laboratory investigations is to achieve clinical applications. In clinical studies, patients are commonly divided into two groups. Patients in control group receive conventional chemotherapy. Patients in treatment group receive conventional chemotherapy plus natural product(s). Hu and coworkers [58] investigated the effect of* Fritillariae thunbergii* in 90 acute leukemia patients and found that the patients in the treatment group had lower number of leukemia cells in bone marrow, decreased protein level of MDR1, and decreased remission rate, compared with patients in the control group. Li and coworkers [59] investigated the effect of the* Fritillariae thunbergii* in 30 patients with acute leukemia patients who expressed high level of MDR1. The patients in the treatment group had lower protein level of MDR1 (3 times lower), higher therapy response rate (20% versus 55%), and lower percentage of leukemic cells in bone marrow (50% versus 26%). Zhao and coworker [60] recruited 36 patients with acute leukemia, who were positive to MRP protein. They performed real-time PCR to quantify bone marrow cell mRNA levels of MRP and *β*2M, and patients with MRP/*β*2M value equal to or greater than 0.3 were considered as MRP positive. The patients treated with ligustrazine injection (120 mg iv. daily for 15 days) were more likely to turn negative for MRP (45.5 versus 7.1%) and have lower percentage of leukemic cells in bone marrow (21.4 versus 55.6%). Some other clinical studies and benefits are included in Table 6. The compositions of formulas are shown in Table 7.

## 7. Conclusion

Drug resistance explains a big part of failure in cancer chemotherapy. Despite the efforts to overcome cancer cell drug resistance, there is no satisfactory solution so far. Natural products provide a vast pool for screening for drugs including candidate compounds to overcome drug resistance. Although the studies included in this review are not particularly rich in scientific details, they show that many natural products, used alone or in combination, are potent inhibitors of drug resistance under laboratory conditions and in clinical setting, which provides informative original observations (including many first time reports) for the scientific and industrial communities. Some single compounds and herbal ones as well as all of the herbal formulas have already been used clinically in treating various diseases including cancer. Future studies may identify natural products for clinical management of cancer cell drug resistance.

## Figures and Tables

**Table 1:** Natural products that can overcome drug resistance without mechanistic information.

Compound name	Dose	Cell model	Chemodrug	IDS^1^	Reference
Single compound					
4′-Methylether-scutellarein	20 *μ*g/mL	JAR/VP16	Etoposide	5.0	[61]
Dauricine	2.5 *μ*g/mL	K562/ADM	Doxorubicin	5.0	[62]
Dihydroartemisinin	0.3 *μ*M	A549/CDDP	Cisplatin	12.3	[63]
Ginsenoside Rh2	40 *μ*M	MCF/ADM	Doxorubicin	31.1	[64]
Isorhynchophylline	1.5–3 *μ*g/mL	A549/DDP	Various	7.2–24.3	[65–67]
Ligustrazine	2.6–10 *μ*g/mL	K562/ADM	Doxorubicin	6.0–178	[68, 69]
Matrine	50 *μ*g/mL	K562/ADM	Doxorubicin	2.2	[70]
Peiminine	20 *μ*g/mL	SGC7901/VCR	Various	6–27	[71]
Schizandrol A	25 *μ*M	HL60/ADR	Daunorubicin	8	[72]
Tanshinone II A	0.2 *μ*g/mL	MCF/ADM	Doxorubicin	2.5	[73]
Single herb extract					
*Eleutherococcus senticosus*	200 *μ*g/mL	K562/ADR	Daunomycin	4.2	[74]
*Andrographis paniculata*	2.4 *μ*g/mL	CHT8/5FU	Various	2.1–2.2	[75]
Indigowoad root	31 *μ*g/mL	BEL7404/ADM	Doxorubicin	4.8	[76]
Formula extract					
Danggui Buxue recipe	300 *μ*g/mL	SGC7901/ADR	Doxorubicin	2.8	[77]
Fufang Jinzi recipe	5 *μ*g/mL	K562/VCR	Vincristine	3.6	[78]

^1^IDS, increase in drug sensitivity (*x*-fold).

IDS = IC50 in control group/IC50 in experimental group.

**Table 2:** Natural products that can overcome drug resistance and inhibit drug transporters.

Compound name	Dose	Drug target^1^	Cell model	Chemodrug	IDS^2^	Reference
Single compound						
Apigenin	15 *μ*M	MDR1	HCT/5FU	5-Fluorouracil	5.8	[79]
Arsenic Trioxide	0.15–0.4 *μ*M	MDR1 and MRP	Various	Various	2.1–3.0	[80–82]
Artemisinin	40 *μ*M	MDR1	MCF7/ADR	Doxorubicin	3.6	[83]
Artesunate	20 *μ*g/mL	MDR1	SP2/0/ADM	Doxorubicin	4.2	[84]
Baicalin	20–300 *μ*g/mL	MDR1 and MRP1	Various	Doxorubicin	6.3–7.8	[85, 86]
Berbamine	20 *μ*M	MDR1	MCF7/ADR	Doxorubicin	28.3	[87]
Berberine	20 *μ*M	MDR1	HCT8/VCR	Vincristine	3.6	[88]
Carnosic acid	5 *μ*g/mL	MDR1	K562/A02	Doxorubicin	12.1	[89]
Chelerythrine	0.1 *μ*M	MDR1	MCF7/Taxel	Paclitaxel	5.9	[90]
Curcumin	2.5–25 *μ*M	MDR1	Various	Various	4.6–4.8	[91, 92]
Elemene	4–18 *μ*g/mL	MDR1 and MRP	Various	Various	3.1–4.1	[93–95]
Ephedrine	75 *μ*g/mL	MDR1	K562/A02	Daunorubicin	5.7	[96]
Epigallocatechin gallate	2–80 *μ*M	MDR1 and ABCG2	Various	Various	2.7–24.2	[97, 98]
Gambogic acid	4 *μ*g/mL	MDR1	SW480/L-OHP	Oxaliplatin	3.7	[99]
Ginsenoside Rb1	80 *μ*M	MDR1	HL60/VCR	Vincristine	2.7	[100]
Ginsenoside Rg3	10–40 *μ*g/mL	MDR1, MRP, and LRP	Various	Various	7.3–11	[101, 102]
Neferine	10–20 *μ*M	MDR1 and MRP	SGC7901/VCR	Various	3.3–43.8	[103–105]
Oridonin	3 *μ*M	MDR1	K562/A02	Various	9.2	[106]
Oxymatrine	0.8 mg/mL	MDR1	MCF7/ADM	Doxorubicin	3.3	[107]
Peimine	400 *μ*g/mL	LRP	A549/DDP	Cisplatin	3.7	[108]
Psoralen	20 *μ*g/mL	MDR1	MCF7/ADR	Doxorubicin	11.8	[109]
Quercetin	Various	MDR1 and MRP1	Various	Various	2.3–22.0	[110, 111]
Sodium norcantharidate	5 *μ*g/mL	MDR1 and MRP	A549/DPP	Cisplatin	2.0	[112]
Tetramethylpyrazine	200–320 *μ*g/mL	MDR1	Various	Doxorubicin	2.1–5.2	[113–115]
Tetrandrine	1 *μ*M	MDR1	Hep3B/ADM	Doxorubicin	12.7	[116]
Single herbal extract						
*Brucea Javanica*	55.3–125 *μ*g/mL	MDR1 and MRP	Various	Various	2.1–5.8	[117, 118]
Cinobufacini	?	MDR1 and MRP1	Raji/ADR	Doxorubicin	255.7	[119]
Grape seed polyphenols	2.4–6.0 *μ*g/mL	MDR1	Various	Various	3.4–4.5	[120, 121]
Hyaluronate Oligomers	100 *μ*g/mL	MDR1 and MRP	MCF7/ADM	Doxorubicin	2.0	[122]
Jew ear	8–10 *μ*g/mL	MDR1 and MRP	Various	Various	2.2–5.6	[123–125]
*Radix notoginseng*	200 *μ*g/mL	MDR1	Various	Various	5.6	[126, 127]
*Rhizoma pinelliae*	6.5 *μ*g/mL	MDR1	K562/A02	Doxorubicin	3.6	[128]
Realgar	25 *μ*g/mL	MDR1	MCF7/ADM	Doxorubicin	2.8	[129]
*Thallus laminariae*	70–78 *μ*g/mL	MDR1	Various	Doxorubicin	4.7–5.0	[130, 130]

^1^Levels of protein or mRNA or both were quantified.

^2^IDS, increase in drug sensitivity (*x*-fold).

IDS = IC50 in control group/IC50 in experimental group.

**Table 3:** Natural products that can overcome drug resistance and inhibit apoptosis signaling.

Compound name	Dose	Drug target^1^	Model cell	Chemodrug	IDS^2^	Reference
Single compound						
Curcumin	10 *μ*M	Caspase-3	HCT8/5FU	5-Fluorouracil	10.1	[131]
Dihydroartemisinin	230 nM	Bcl-2, Bax, and Caspase-3	A549/DDP	Cisplatin	8.3	[132]
Elemene	10–30 *μ*g/mL	GSH, Bcl-2, and Bad	Various	Various	2.2–3.7	[133–135]
Emodin	30 *μ*M	p-EGFR and p-ERK	HCC827/GR	Gefitinib	6.0	[136]
Epigallocatechin gallate	80 *μ*M	Bcl-2 and Bax	K562/A02	Doxorubicin	24.2	[137]
Ginsenoside Rh2	10 *μ*M		A549/DDP	Cisplatin	3.5	[138]
Neferine	10 *μ*M	Bcl-2	SGC7901/VCR	Vincristine	43.8	[139]
Parthenolide	20 *μ*M	Survivin and Bcl-2	A549/GR	Gefitinib	8.7	[140]
Phenylpropanoid glycoside	40 *μ*g/mL	Caspase-3	LoVo/ADR	Doxorubicin	9.9	[141]
Retinoic acid	1 *μ*M	Bcl-2	MCF7/TAM	Tamoxifen	6.7	[142]
Tetrandrine	1.0 *μ*g/mL	Bcl-2 and Bax	BIU-87/ADM	Doxorubicin	2.2	[143]
Herbal extract						
Realgar	15 *μ*g/mL	Bcl-2	MCF7/ADM	Doxorubicin	2.3	[144]

^1^Levels of protein or mRNA or both were quantified.

^2^IDS, increase in drug sensitivity (*x*-fold).

IDS = IC50 in control group/IC50 in experimental group.

**Table 4:** Natural products that can overcome drug resistance and regulate multiple pathways.

Compound name	Dose	Pathways^1^	Cell model	Chemodrug	IDS^2^	Reference
Single compound						
Arsenic trioxide	0.8 *μ*M	MDR1 and GST-*π*	SGC7901/ADR	Doxorubicin	2.1	[82]
Berbamine	20 *μ*M	MDR1 and survivin	K562/A02	Doxorubicin	41.2	[145]
Carnosic acid	25 *μ*M	MDR1 and Bcl-2	K562/A02	Doxorubicin	12.1	[146]
Curcumin	25 *μ*M	MRP and Bcl-2	HL60/ADR	Doxorubicin	4.2	[147]
Emodin	10 *μ*M	MRP1, Topo II b, GST-*π*, and Bcl-2	HL60/ADR	Doxorubicin	4.1	[148]
Honokiol	6.5 *μ*g/mL	MDR1 and NFkB	U937/ADR	Doxorubicin	2.2	[149]
Methylether-scutellarein	20 *μ*g/mL	ABC and apoptosis genes	JAR/VP16	Various	2.5–5.0	[150]
Quercetin	10 *μ*g/mL	MDR1 and survivin	A549/DDP	Cisplatin	3.5	[151]
Quercetin	40 *μ*M	ABC, Bcl-2, and SLC genes	K562/A	Doxorubicin	3.7	[152]
Quercetin	40 *μ*M	MDR1, MRP, GST-*π*, and H-ras	Bel-FU	5-Fluorouracil	2.4	[153]
Schizandrin B	10 *μ*M	MDR1, pPI3K, and pAKT	U-2 OS/ADR	Various	2.3–2.9	[154]
Schizandrin A	50 *μ*M	MDR1 and GSH	Various	Various	41.2–147.5	[155]
Sodium selenite	10 *μ*M	MDR and Bcl-2	K562/ADR	Doxorubicin	2.3	[156]
Tetrandrine	1 *μ*g/mL	MDR1, survivin, and caspase-3	BIU87/ADM	Doxorubicin	5.3	[157]
Herbal extract						
*Radix bupleuri*	300 *μ*g/mL	MDR1 and TopoII*α*	Bel-7402	Verapamil	15.6	[158]
Grape seed polyphenols	6 *μ*g/mL	MDR1 and Bcl-2	GBC/SD	Various	3.4–4.5	[159]
Tea polyphenols	7.5 *μ*g/mL	MRP and Bcl-2	HL60/VCR	Various	2.3–9.1	[160]

^1^Levels of protein or mRNA or both were quantified.

^2^IDS, increase in drug sensitivity (*x*-fold).

IDS = IC50 in control group/IC50 in experimental group.

**Table 5:** Natural products overcome drug resistance in serum pharmacology studies.

Name	Dose (days)	Serum dose^1^	Cell model	Chemodrug	IDS^2^	Reference
Buzhong Yiqi Tang	5.7 g/kg (3)	15	A549/DDP	Cisplatin	3	[161]
Jiedu Huayu recipe	3 g/kg (3)	10	HL60/Adr	Doxorubicin	8.4	[162]
*Scutellariae barbatae*	14.1 g/kg (4)	0.5	K562/A02	Various	2.2–5.4	[163]
Shehuang Xiaoliu	29.7 g/kg (10)	20	Bel7402	Doxorubicin	8.3	[164]
Zedoary turmeric oil	0.8 mg/kg (3)	20	SGC7901/CDDP	Verapamil	6.1	[165]

^1^Percentage of serum being tested in addition to the conventional 10% of fetal bovine serum in cell culture.

^2^IDS, increase in drug sensitivity (*x*-fold).

IDS = IC50 in control group/IC50 in experimental group.

**Table 6:** Clinical studies and benefits.

Disease (number of patients)	Treatment agent	Clinical benefit	Reference
Relapsed non-Hodgkin's lymphoma (60)	Ligustrazine	Higher overall response rate	[166]

Breast cancer (76)	Chinese magnolcavine fruit	Increase in response rate, delayed remission, and increase in the MRP negativity rate	[167]

Advanced gastrointestinal cancer (54)	Changweiqing formula	Higher response rate and Karnofsky score Longer survival time Lower level of MDR1 in blood cells	[168]

Gastric cancer, stages II-III (30)	Didang formula	Higher response rate and better life quality Lower level of MDR1 in blood cells	[169]

Liver cancer (71)	Xiaochaihu formula	Higher response rate and lower protein level of MDR1 in cancer tissue	[170]

Gastric cancer, stages II-III (60)	Shenqi Jianwei formula	Higher response rate, lower level of lower of CEA, and higher Karnofsky score	[171]

Breast cancer (60)	Pingxiao formula	Higher response rate and lower protein levels of MDR1 and GST*π* in cancer tissues	[172]

Breast cancer (53)	Fukangling capsule	Higher response rate and lower protein levels of MDR1 and GST*π* in cancer tissues	[173]

Non-small cell lung cancer (60)	Qiankun capsule	Lower level of blood TGF-*α* and lower number of PCNA positive cells as well as lower protein levels of LRP and MRP in cancer tissue	[174]

Breast cancer, stages II-III (33)	Fukangling capsule	Higher response rate and lower protein level of MDR1 in cancer tissue	[174]

**Table 7:** Major composition of formulas.

Name of formula	Major composition
Changweiqing	*Astragalus membranaceus, Atractylodes macrocephala Koidz, Radix Codonopsis Pilosulae, Polyporus, Coix chinensis Tod, Fructus Akebiae Trifoliatae, Vitis quinquangularis Rehd, *and* Sargentodoxa cuneata *(Oliv.) Rehd. et Wils

Wenxia Fang	*Rhizoma et Radix Rhei Palmat, Aconitum carmichaeli Debx, Panax ginseng *C. A. Mey, and *Angelica sinensis*

Buzhong Yiqi	*Astragalus membranaceus, Radix Glycyrrhizae, Panax ginseng *C. A. Mey*, Angelica sinensis, Pericarpium Citri Reticulatae, Cimicifuga foetida *L.*, Radix Bupleuri, *and* Atractylodes macrocephala Koidz*

Jiedu Huayu	*Indigo Naturalis, Asarum sagittarioides *C. F. Liang*, Rhizoma Paridis Chinensis, Curcuma zedoaria * (Christm.) Rosc.*, Rhizoma Chuanxiong, Radix Salviae Miltiorrhizae, *and* Fructus Psoraleae*

Shehuang Xiaoliu	*Moschus berezovskii Flerov, Bos taurus domesticus Gmelin, Curcuma zedoaria * (Christm.) Rosc.*, Astragalus membranaceus, Radix Codonopsis Pilosulae, Scutellaria barbata *D. Don., and *Hedyotis diffusa*

Didang	*Hirudo, Tabanus, Semen Persicae, *and* Rhizoma et Radix Rhei Palmat*

Xiaochaihu	*Viola philippica *Car.*, Hedyotis diffusa, Pyrrosia lingua *(Thunb.)* Farwell, Zaojiaoci (Spina Gleditsiae), Manis pentadactyla Linnaeus, *and* Smilax glabra *Roxb.

Shenqi Jianwei	*Radix Codonopsis Pilosulae, Astragalus membranaceus, Atractylodes macrocephala Koidz, Angelica sinensis, Radix Paeoniae Alba, Smilax china *L.*, Taraxacum mongolicum, Crataegus pinnatifida, Perilla frutescens * (L.) Britton.*, Radix Aristolochiae, *and* Pericarpium Citri Reticulatae*

Pingxiao	*Curcuma longa *L.*, Agrimonia pilosa *Ledeb.*, Trogopterus xanthipes *Milne-Edwards*, Alumen, Nitrum, Toxicodendron vernicifluum *(Stokes) F. A. Barkl.*, Bitter Orange Fructus Aurantii, *and* Strychnos nux-vomica *Linn.

Fukangling capsule	*Panax ginseng *C. A. Mey*, Astragalus membranaceus, Ganodermae Lucidi, Rhizoma Polygonati, Radix Glehniae, *and* Fructus Schisandrae Chinensis *

Qiankun capsule	*Radix Codonopsis Pilosulae, Radix Rehmanniae, Astragalus membranaceus, Fructus Psoraleae, Furctus Lycii barbari, Fructus Ligustri Lucidi, Asarum sagittarioides *C. F. Liang*, Rhizoma Dioscoreae Bulbiferae, Indigo Naturalis, Herba Cirsii Setosi, Panax notoginseng, Lobed Kudzuvine Root, *and* Bos taurus domesticus Gmelin*

Huatan Sanjie	*Smilax china, Rhizoma pinelliae, Bulbus Fritillariae Thunbergii, Coix chinensis Tod, Pollen, *and* Curcuma zedoaria *(Christm.) Rosc.
